# Semi-automatic segmentation of the fetal brain from magnetic resonance imaging

**DOI:** 10.3389/fnins.2022.1027084

**Published:** 2022-11-11

**Authors:** Jianan Wang, Emily S. Nichols, Megan E. Mueller, Barbra de Vrijer, Roy Eagleson, Charles A. McKenzie, Sandrine de Ribaupierre, Emma G. Duerden

**Affiliations:** ^1^Biomedical Engineering, Western University, London, ON, Canada; ^2^Applied Psychology, Faculty of Education, Western University, London, ON, Canada; ^3^Western Institute for Neuroscience, Western University, London, ON, Canada; ^4^Department of Obstetrics and Gynaecology, Schulich School of Medicine & Dentistry, Western University, London, ON, Canada; ^5^Department of Electrical and Computer Engineering, Western University, London, ON, Canada; ^6^Department of Medical Biophysics, Schulich School of Medicine & Dentistry, Western University, London, ON, Canada; ^7^Department of Clinical Neurological Sciences, Schulich School of Medicine & Dentistry, Western University, London, ON, Canada; ^8^Department of Anatomy and Cell Biology, Schulich School of Medicine & Dentistry, Western University, London, ON, Canada

**Keywords:** fetal, MRI, brain, linear registration, nonlinear registration, volumetric reconstruction

## Abstract

**Background:**

Volumetric measurements of fetal brain maturation in the third trimester of pregnancy are key predictors of developmental outcomes. Improved understanding of fetal brain development trajectories may aid in identifying and clinically managing at-risk fetuses. Currently, fetal brain structures in magnetic resonance images (MRI) are often manually segmented, which requires both time and expertise. To facilitate the targeting and measurement of brain structures in the fetus, we compared the results of five segmentation methods applied to fetal brain MRI data to gold-standard manual tracings.

**Methods:**

Adult women with singleton pregnancies (*n* = 21), of whom five were scanned twice, approximately 3 weeks apart, were recruited [26 total datasets, median gestational age (GA) = 34.8, IQR = 30.9–36.6]. T2-weighted single-shot fast spin echo images of the fetal brain were acquired on 1.5T and 3T MRI scanners. Images were first combined into a single 3D anatomical volume. Next, a trained tracer manually segmented the thalamus, cerebellum, and total cerebral volumes. The manual segmentations were compared with five automatic methods of segmentation available within Advanced Normalization Tools (ANTs) and FMRIB’s Linear Image Registration Tool (FLIRT) toolboxes. The manual and automatic labels were compared using Dice similarity coefficients (DSCs). The DSC values were compared using Friedman’s test for repeated measures.

**Results:**

Comparing cerebellum and thalamus masks against the manually segmented masks, the median DSC values for ANTs and FLIRT were 0.72 [interquartile range (IQR) = 0.6–0.8] and 0.54 (IQR = 0.4–0.6), respectively. A Friedman’s test indicated that the ANTs registration methods, primarily nonlinear methods, performed better than FLIRT (*p* < 0.001).

**Conclusion:**

Deformable registration methods provided the most accurate results relative to manual segmentation. Overall, this semi-automatic subcortical segmentation method provides reliable performance to segment subcortical volumes in fetal MR images. This method reduces the costs of manual segmentation, facilitating the measurement of typical and atypical fetal brain development.

## Introduction

Magnetic resonance imaging (MRI) of the fetal brain for clinical purposes has advanced considerably in recent years due to its application in assessing atypical brain development and brain injury and its potential utility in predicting functional outcomes in high-risk fetuses (Banović et al., 2014; Brossard-Racine et al., 2014, 2019; Cesaretti et al., 2016; Andescavage et al., 2017). Additionally, research-based MRI studies of typical fetal brain development have provided important normative data for subsequent comparison with clinical populations (De Asis-Cruz et al., 2021). MRI methods for the characterization of fetal brain abnormalities are of key clinical relevance due to the high incidence of central nervous system malformations (i.e., anencephaly, ventriculomegaly, schizencephaly, and callosal agenesis) in as many as 1/1,000 fetuses (Werner et al., 2018). In particular, detection of delayed brain growth offers new opportunities to identify objective biomarkers that can facilitate a better understanding of fetal brain development, improved management of high-risk pregnancies (Rutherford et al., 2008; Cesaretti et al., 2016; Knezović et al., 2019; Wu et al., 2020), and potentially early detection of neurodevelopmental outcome (Banović et al., 2014; Bonnet-Brilhault et al., 2018). Additionally, longitudinal studies point to fetal brain abnormalities as an important contributor to later life neurodevelopmental and psychiatric disorders (Thomason, 2020). Better understanding of typical fetal brain developmental trajectories may aid in predicting functional outcomes.

Quantitative measurements of the fetal brain and subcortical volumes can support characterizing normal brain development and identifying early predictors of brain dysmaturation (Boardman et al., 2010; Rathbone et al., 2011). However, the traditional manual segmentation of MR images is time-consuming and requires high-level expertise; thus, it is impractical to implement these methods to large datasets. For functional imaging, manual segmentation of 4D fetal images can take upwards of 30 h to complete a single scanning run in an individual participant’s data (Nichols et al., 2022). Automatic segmentation pipelines and routines developed for neonatal and child imaging protocols are not appropriate for studying fetal brain tissue due to the variations in image acquisition, and maturational differences leading to poorer contrast of the gray and white matter. Therefore, reliable automatic segmentation methods for fetal MR images are needed to study typical and atypical fetal brain development.

We applied two atlas-based segmentation techniques, linear and nonlinear atlas registration algorithms, to perform the regional segmentation of the cortex and subcortical areas in the fetal brain to examine their macrostructural development. The cerebellum and thalamus are key deep brain structures related to alterations in neuro-cognition and motor behaviors that are typically seen in infants impacted by growth restriction as well as preterm birth. Early growth impairments or alterations in the trajectory of growth in the cerebellum have been found to be associated with an increased risk of autism (Beversdorf et al., 2005; Limperopoulos et al., 2007). Further, cerebellar lesions in adulthood can impair decision-making, working memory, and planning (Koziol et al., 2014; Clausi et al., 2015). Deficits in linguistic abilities, anxiety, and impaired social behavior have also been associated with cerebellar lesions (Schmahmann, 2004; Ramphal et al., 2021). Early cerebellar lesions at the vermis area can produce impaired eye gaze, anxiety, and lack of mental flexibility such as stereotyped behavior (Wells et al., 2008; Clausi et al., 2015). The thalamus, is the primary relay station to the cortex and plays an important role in motor and cognitive functions (Dehghani and Wimmer, 2019). Atypical development of the thalamus is associated with impaired emotional processing, language, and social cognition in children and adult populations with neurodevelopmental disorders (Hardan et al., 2006). Volumetric segmentations of the cerebellum and thalamus can aid in morphological analysis of the growth of the two brain structures, which may be beneficial to exploring *in utero* origins of cognitive and motor functions in the typically developing fetus.

Manual based segmentation methods have been employed to segment subcortical fetal brain tissues (Twickler et al., 2002). However, these methods are very time consuming and require high level expertise. Deep-learning methods such as deep convolutional neural networks (CNNs) have been used to segment subcortical structures in fetal MR images (Khalili et al., 2019; Zhao et al., 2022). Atlas-based segmentation methods have been used to target deep brain structures in fetal MR images (Habas et al., 2010). Landmark-based rigid image transformation has been applied to fetal MR images to obtain volumetric and cortical measures (Wu et al., 2020). However, deformable registrations may be more robust. They may be able to more accurately segment subcortical structures in fetal MR images compared to linear registration, but are more computationally intensive and may be more challenging to implement in clinical settings. Using an atlas-based method, we examined whether more computationally intensive deformation image registration methods, using the Advanced Normalization Tools (ANTs), are needed for adequate subcortical segmentation compared to an affine image registration FLIRT (FMRIB’s Linear Image Registration Tool). This research aimed to develop and implement a semi-automatic pipeline combining semi-automatic fetal brain reconstruction, segmentation, volumetric reconstruction, and atlas registration algorithms for subcortical segmentation in fetal brains to extract and analyze subcortical volumes.

## Materials and methods

### Participants

Pregnant individuals with singleton fetuses were recruited to the study. All participants self-identified as native English speakers and reported no history of psychiatric illness, neurological disorder, or hearing impairment.

The study was approved by the Health Sciences Research Ethics Board at Western University. The research was conducted according to the principles expressed in the Declaration of Helsinki. The letter of information was sent to participants in advance of the study, and a member of the research team reviewed the protocol. All participants provided informed consent.

### Magnetic resonance imaging protocol

Participants were scanned at two sites, and the study procedures were maintained at both locations. The majority of the scans (*n* = 21) were acquired on a 3T MRI [General Electric (GE), Milwaukee, WI, SA; MR7500] with a 32-channel GE torso coil and a 60 cm bore at the Translational Imaging Research Facility at the Robarts Research Institute. Of the 21 scans, 5 were repeat scans whereby the mothers returned for an identical scanning session. The other five scans were collected on a 70 cm bore 1.5T (GE, MR450w) with a GEM posterior and anterior array coil at London Health Science Center.

**FIGURE 1:**
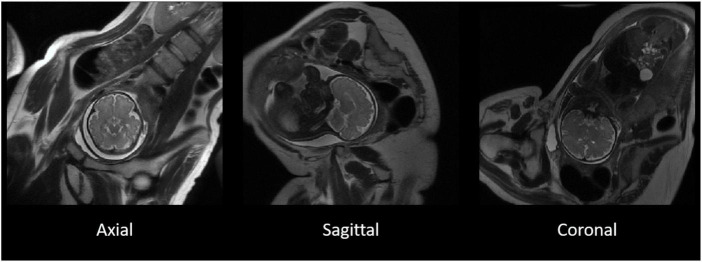
The original T2-weighted acquisition of a fetal MR image in axial, sagittal, and coronal planes. T2-weighted images acquired separately in three separate image planes in the axial **(left)**, sagittal **(middle)**, and coronal **(right)** in a representative participant. The three image planes were subsequently used for the reconstruction of 3D images.

The T2-weighted MR images were acquired using a single shot fast spin echo (SSFSE) sequence [repetition time (TR) > 1,200 ms, echo time (TE): 81.36–93.60 ms, voxel size: 0.98 mm × 1.96 mm × 8 mm and 0.125 mm × 0.17 mm × 9 mm], applied in three image planes (Figure 1).

### Volumetric reconstruction of magnetic resonance images

NiftyMIC (Ebner et al., 2020) was used for fetal brain segmentation and 3D reconstruction. The main processing pipeline for detection and segmentation of the fetal brain included with NiftyMIC involves only a single command (*fetal_brain_seg*) and can be executed unsupervised. Various features of different slice-to-volume reconstructions methods including NitfyMIC have been compared for fetal MRI, and have reported comparable results (Payette et al., 2021).

It was essential to first estimate the fetal brain location in the MR image such that a bounding box was created to reduce both unrelated contents and image space, as well as the algorithm processing time for the later more precise fetal brain segmentation algorithm using 2D P-Net CNN (Yamashita et al., 2018). NiftyMIC’s *fetal_brain_seg* command was then executed on the MR image, generating a mask of the fetal brain in the surrounding tissue for each slice within the image. This step took under 2 min per stack of 2D slices.

The resulting masks were then reviewed using FSLeyes.^1^ These automatically generated 2D fetal brain masks from NiftyMIC were suboptimal for most participants, resulting in either over- or under-estimating fetal brain tissue in the slices; surrounding maternal gray and white tissue were still evident in the slices, depending on the acquisition and field of view. Therefore, manual adjustments of the masks, such as filling and excluding pixels, were performed on all automatically generated 2D masks (*n* = 26). Time spent manually editing ranged from 1 to 15 min per stack of 2D slices, with the majority taking under 5 min to complete.

After segmenting fetal brains in the 2D planes, the stacks of 2D slices were reconstructed into 3D volumes, and the 2D fetal brain segmentations were also reconstructed into 3D space. The 2D MR image slices could be corrupted by low-frequency bias field signals to blur the high-frequency contents, such as edges and contours. Intensity variance also resulted from existing bias field signals where the same tissue had a uniformed pixel gray level in the images. Thus, the stacks of segmented 2D fetal brain slices were first bias-field corrected. Second, the bias-field corrected 2D slices were reconstructed into a 3D volume by the slice-to-volume process that rigidly registered the 2D slices to one randomly selected target slice from the fetal brain MR images so that all the slices were volumetrically aligned. The slice-to-volume process also used linear regression to correct and match the slices’ voxel intensities to the target slice’s voxel intensity. Third, the volume-to-volume process was performed on the 2D slices and previously segmented 2D masks to reconstruct into 3D volumes and 3D fetal brain masks in native space. Processing times varied but were up to 2 h in some participants’ data (Uus et al., 2022).

Subsequently, the native-space 3D volumes were rigidly registered to a spatiotemporal atlas developed from images acquired at 3T MRI from typically developing fetuses to obtain the volumetric reconstruction in the standard anatomical planes of atlas space.

#### Registration-based subcortical segmentation

**FIGURE 2:**
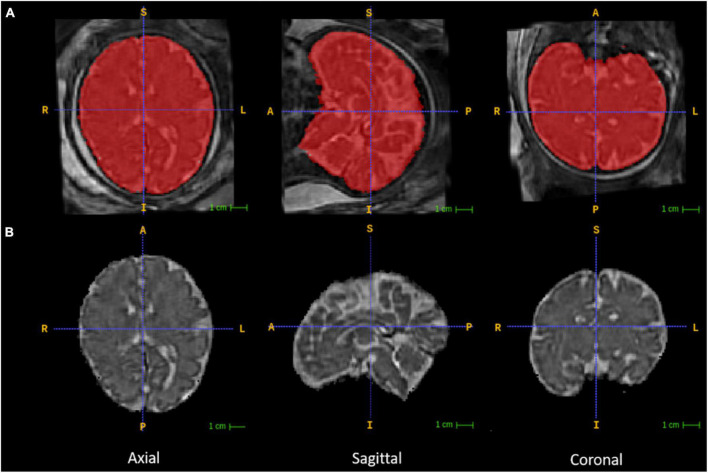
Fetal brain segmentation. Row **(A)** includes the volumetrically reconstructed fetal brains in three planes. The red areas are the manually segmented fetal brain binary masks. Row **(B)** includes the orientation-corrected and skull-stripped (using the binary masks in red) fetal brain volumes in three planes.

The reconstructed 3D fetal brain masks were applied onto the reconstructed 3D brain volumes for fetal brain skull stripping (Figure 2). The 3D brain volumes were segmented with the binary masks for fetal brain-only MR images. This segmentation was a prerequisite for later subcortical segmentation utilizing image registration since image registration for tissue alignment assumes the target object and the moving object are the same tissue with similar shapes. Registering the skull-stripped fetal brain atlas to the subject’s fetal brain, excluding maternal tissue, would reduce unrelated content for meaningful registration results. The skull stripping step was performed using 3dcalc from the AFNI toolkit that multiplied the reconstructed 3D fetal brain image with the binary 3D masks. Then the orientations of the skull-stripped MR images were manually adjusted according to the orientations of the age-appropriate fetal brain atlas using the ITK-SNAP GUI (Figure 2; graphical user interface^2^).

**FIGURE 3:**
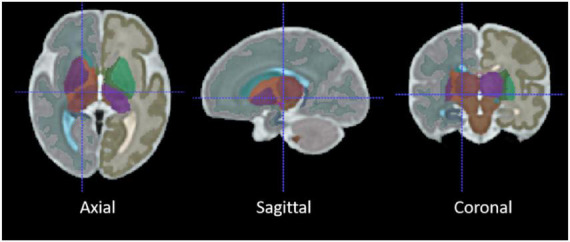
The average 36-week GA fetal brain atlas, including cerebellum and thalamus labels. The axial, sagittal, coronal, and 3D rendered views of the age-appropriate fetal brain atlas whereby deep brain tissues are color-coded.

Two different registration toolkits were applied to the reconstructed images and compared to determine an optimal fetal subcortical segmentation strategy. Deformable registration was performed using ANTs (Avants et al., 2008) using the well-known SyN (symmetric image normalization) method, and linear (affine) atlas registration was performed using FLIRT (Jenkinson et al., 2012). The fetal brain atlas (Figure 3; Gholipour et al., 2017) is an averaged atlas from fetuses imaged at 36 weeks GA with predefined labels of deep-brain structures, including the thalamus and cerebellum. The atlas was nonlinearly and linearly registered into the native participant 3D MRI space. The transformation matrix was saved and applied onto the atlas mask to warp the tissue labels into subject space. The transformed atlas labels were used as thalamus and cerebellum masks and were compared with manual masks by calculating DSCs for the reliability test.

The applied FLIRT registration tool implemented the correlation ratio similarity metric for linear (affine) registration as the default parameters. The ANTs registration tool used a mutual information (MI) similarity metric for linear (rigid and affine) registration and nonlinear (SyN) registration. Different combinations of similarity metrics for both linear and nonlinear image registration of ANTs were also applied and compared to find the more suitable image registration method for our MR image data. The cross-correlation (CC) and MI similarity metrics, provided in the ANTs toolbox, which are both sufficient for intra-modality registration were used for rigid, affine, and SyN registration algorithms.

The FLIRT linear image registration was performed using the command line tool with the DOF (degree of freedom) option set at 12. The ANTs linear image registration (12 DOF) was performed using the antsRegistration command line tool by defining the rigid and affine transformations. The ANTs nonlinear registration (millions of DOF) algorithm using the MI metric was performed using the default antsRegistraionSyNQuick.sh script. Keeping every other parameter the same as the antsRegistraionSyNQuick.sh script, the ANTs nonlinear registration using the CC metric was also performed using the antsRegistration command line tool by adding the SyN transformation definition upon the linear registration parameters. To apply the transformation matrices to the atlas masks, the FLIRT command line tool was defined with the applyxfm option, and the ANTs command line tool was antsApplyTransformations. The whole fetal brain, cerebellum, and thalamus volumes were computed from the skull-stripped fetal brain masks and subcortical masks.

#### Manual subcortical segmentation protocol

Anonymized with respect to GA, the left and right thalamus and cerebellum were delineated in all reconstructed T2-weighted images. The 3D reconstructed T2-weighted images were visualized and segmented using ITK SNAP. The displays provided simultaneous coronal, sagittal and axial views of the brain and created a 3D image of the thalamus and cerebellum. Bilateral thalamus and cerebellum masks were created through the visual identification and tracing of these brain regions in each slice. A three-step segmentation protocol was applied to each image to segment the cerebellum and thalamus. The thalamus was segmented first, followed by the cerebellum. In each scan, the thalamus was present in approximately 40 slices, whereas the cerebellum was present in approximately 50 slices. Segmentations were based on the intensity differences between white and gray matter.

*Step 1: Segmentation of the cerebellum and thalamus*. Dependent on the participants and the resolution of the images, the rater segmenting the images manually composed segmentations through all three viewpoints (sagittal, coronal, and axial) to ensure that the masks were accurate in all viewpoints. The initially completed segmentations were verified in the other views, and any incorrectly identified areas were omitted and revised.
*Step 2: Inspection of the 3D surface*. The segmented cerebellum and thalamus masks were represented in a 3D display through ITK-SNAP. The surface of the cerebellum and thalamus is expected to be smooth throughout, so any areas on the masks that protruded excessively were trimmed through a smoothing feature on ITK-SNAP.
*Step 3: Segmentation of left and right hemispheres*. Once complete, cerebellum and thalamus masks were segmented into left and right hemispheres. Each mask was segmented and split into the left and right hemispheres by identifying the brain’s midline. These segmentations were verified across all three viewpoints to ensure accuracy and to revise the original segmentations.

### Protocol reliability testing

Three fetal MR images were randomly selected and re-segmented by the same rater to assess the reliability of the three-step manual segmentation protocol. The re-segmentations of the left and right thalamus and cerebellum in the fetal MR images were performed 6 months after the original segmentations to ensure that the rater’s memory would not unduly influence the results. This type of test-retest metric, intra-rater reliability, can be used as an upper bound metric to assess the accuracy of the segmentations of the thalamus and cerebellum. The protocol’s reliability was measured using the Dice similarity metric, which evaluates the spatial and volumetric overlap of the original and re-segmented label volumes.

### Manually adjusting automatically generated masks from NiftyMIC

Anonymized with respect to GA, whole brain masks were manually segmented in all 25 fetal brain scans. A three-step segmentation protocol (described below) was applied to each image to segment the whole brain masks. The whole brain appeared in approximately 90 slices.

*Step 1: Automatic segmentation*. Whole brain masks were generated automatically for each subject using NiftyMIC software.
*Step 2: Manual segmentation*. Brain masks generated automatically through NiftyMIC were contrasted against the original brain scan for each subject on ITK-SNAP. Each mask was manually edited to ensure that the mask fit the image. Dependent on the subject and the clarity of the image, the individual segmenting the images manually worked through all three viewpoints (sagittal, coronal, and axial) to ensure that the masks were accurate in all viewpoints. The initially completed segmentations were verified in the other views, and any incorrectly identified areas were omitted and revised. Any area of the mask that protruded excessively outside the brain region was removed. Additionally, any areas of the brain that were not covered by the mask were filled in appropriately.
*Step 3: Segmentation of left and right hemispheres*. Once the segmentations were complete, the whole brain masks were segmented into left and right hemispheres. Each mask was segmented and split into the corresponding hemisphere by identifying the midline of the brain. These segmentations were verified across all three viewpoints to ensure accuracy and to revise the original segmentations.

### Software installation and operating system decency

The computer used in this study was built with the 10th generation of intel i7 CPU (central processing unit) with 8 cores and 16 threads, 32 GB of RAM (random access memory). The operating system used for this study was Ubuntu 18.04. ITK-SNAP (version 3.6.0), AFNI (version 20.3.01), convert3d package (version 1.0.0), FSL package (version 6.0.4) including FLIRT was installed locally from source. ANTs was provided by and installed on the SciNet supercomputer center at the University of Toronto (i.e., Digital Research Alliance of Canada). NiftyMIC was installed with the provided Docker image.

### Statistical analysis

The robustness of the entire automatic fetal deep brain structure segmentation workflow was tested by comparing the automatically segmented masks and manually segmented masks by calculating the DSCs of the common areas covered. The DSC, which computes the ratio of two times the common area to the sum of both areas, was calculated using the formula $D=\frac{2\,\left(A\cap B\right)}{A+B}$, where *A* and *B* represent the automatic and manual masks. The masks for the left and right thalamus and cerebellum were combined.

Statistical analyses were performed using SPSS (version 27, Armonk, NY, USA). The resulting DSCs were non-normally distributed, based on Shapiro–Wilk’s tests (all, *p* < 0.02). Therefore, a nonparametric Friedman’s test for repeated measures data was applied to the DSCs. We had a single hypothesis regarding deformable registration methods, so the alpha level was set at *p* < 0.05. The DSCs range from 0, indicating no spatial overlap between the binary segmentation results calculated automatically versus gold-standard manual segmentations, to 1, indicating complete overlap (Cohen, 1960). Moderate overlap occurs when DSCs are 0.5–0.6, while very good overlap occurs at >0.7.

To calculate the DSCs, four regions of interest (ROI): the right cerebellum, left cerebellum, right thalamus, and left thalamus, were extracted from the registration-based subcortical masks using the combination of 3dcalc and 3dcluster command line tools from AFNI. The reason for this step is that the manually drawn subcortical masks of one participant were traced separately for the four ROIs described above. The DSCs were then calculated by overlaying the automatically extracted ROIs from the five different registration methods with the corresponding manual ROIs using the c3d-overlay command line tool of the convert3d package from ITK-SNAP. The c3d command line tool produced the DSCs and redirected the output numbers to print into text files. An in-house Python script was developed to read and write the DSCs from the text files into CSV format.

## Results

### Participants

A total of 21 pregnant adult women participated in the MRI study. Five women returned for a second scan (median time between scans = 3.5 weeks). This resulted in a total of 26 scans that were subsequently used for the analysis. The majority of scans were acquired during the women’s third trimester of pregnancy (*n* = 24, 92%), with the other scans occurring in the near third trimester (range: 27.6–39 weeks of GA). The median GA for all 26 scans was 34.8 weeks (Table 1).

**TABLE 1:** Maternal ages and fetal gestational ages.

Characteristic	Total (*n* = 26)
Maternal ages, median years (IQR)	33.5 (29.3–36)
Fetal gestational age, median weeks (IQR)	34.8 (30.9–36.6)

Ages of the mothers (years) and fetuses (weeks’ gestation), IQR, interquartile range (25%ile–75%ile).

### Two-dimensional fetal brain segmentation and 3D volumetric reconstruction

**FIGURE 4:**
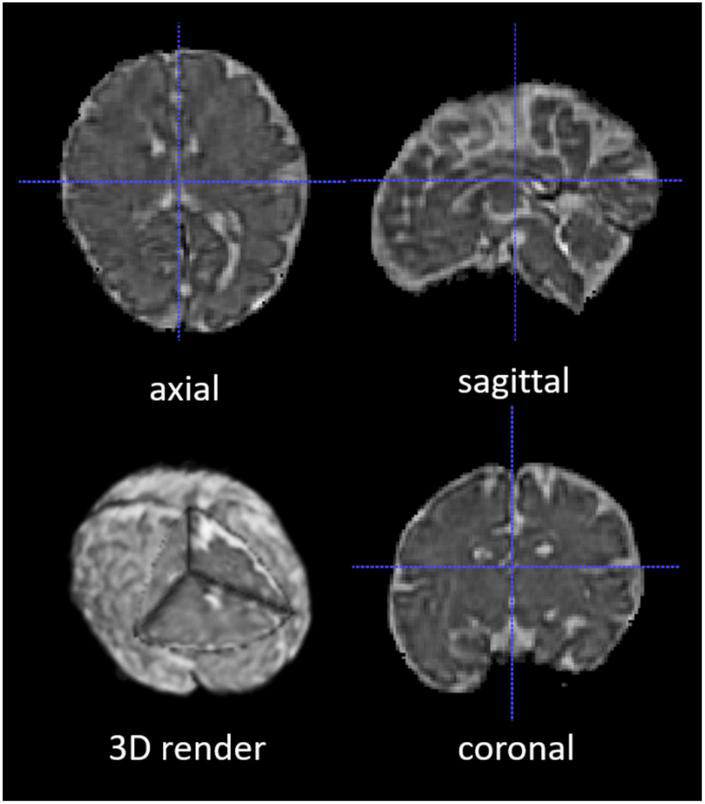
A segmented and volumetrically reconstructed fetal brain image using NiftyMIC. The original 2D slices of fetal MR images were automatically segmented and manually adjusted for fetal brain 2D masks. Then the 2D slices and 2D brain masks were reconstructed into 3D volumes and 3D masks with motion correction. This figure shows an example of the skull-stripped, orientation-adjusted 3D fetal brain volumes in axial, sagittal, coronal, and 3D-rendered views.

The 2D fetal brain masks of the stacks of the original fetal brain MR images were automatically segmented using NiftyMIC in the axial, coronal, and sagittal image planes. For the NiftyMIC volumetric reconstruction algorithm to perform optimally, the 2D auto-masks were manually adjusted using ITK-SNAP for the over- and under-estimations of fetal brain tissue by the NiftyMIC segmentation algorithm. The volumetric reconstruction process was performed on all 26 scans from the 21 total participants. On a total of 25 scans from 20 participants were 2D masks (96%) successfully reconstructed into 3D space (Figure 4). One participant’s data, from the total of 21 participants, was excluded due to a complete failure of the fetal brain segmentation and volumetric reconstruction routine. Once reconstructed, the image dimensions were *X* = 122, *Y* = 127, *Z* = 103 and the voxel size was 1 mm^3^.

Skull stripping and orientation tags correction were successfully applied to the reconstructed 3D volumes. Based on the skull-stripped automatically reconstructed 3D fetal brain MR images, manual segmentations of the thalamus and cerebellum on both left and right sides were successfully performed. The median volumes of the subcortical ROI and whole brain volumes are presented in Table 2, along with the interquartile ranges (IQR). Left and right volumes were combined.

**TABLE 2:** Fetal brain volumes.

Characteristic	Total (*n* = 25)
Cerebellum, median volumes mm^3^ (IQR)	13,365 (10,167–17,783)
Thalamus, median volumes mm^3^ (IQR)	3,850 (2,714–8,381)
Whole brain, median volumes mm^3^ (IQR)	373,186 (285,450–405,289)
GA, median weeks (IQR)	34.6 (30.9–36.4)

GA, Gestational age (weeks), IQR, interquartile range (25%ile–75%ile).

**FIGURE 5:**
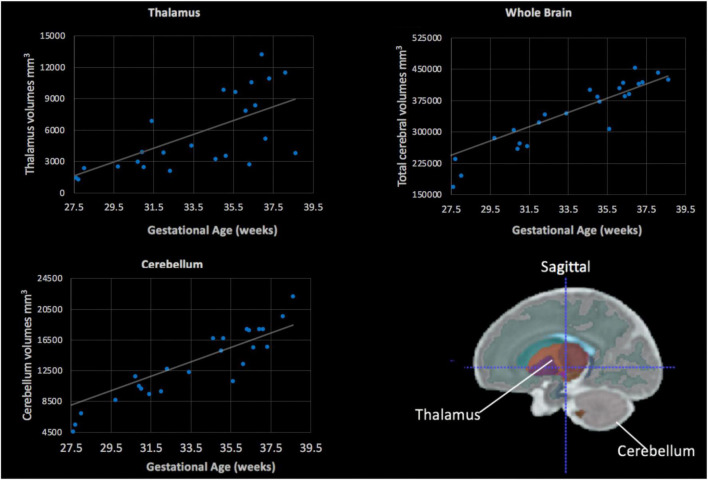
Fetal subcortical volumes (*y*-axis) plotted in relation to gestational age (*x*-axis). Fetal thalamus **(top, left)**, whole brain **(top, right)** and cerebellum **(bottom, left)** volumes were plotted in relation to gestational age in weeks. The cerebellum, thalamus and total cerebral volumes showed a positive linear association with gestational age (all, *p* < 0.05). **(Bottom, right)** The color-coded fetal atlas overlaid on a atlas MRI demonstrates the location of the thalamus (orange/purple) and the cerebellum (gray).

Of the 25 scans, the majority (*n* = 21) were completed on a 3T MRI and 4 were completed at 1.5T. None of the manually segmented volumes for the thalamus, cerebellum or total cerebral volumes differed based on the Tesla strength of the magnets when adjusting for gestational age (all, *p* > 0.05). The averaged left and right thalamus and cerebellum volumes were plotted against gestational age (Figure 5). All regions, the cerebellum (*r* = 0.74, *p* < 0.001), thalamus (*r* = 0.7, *p* < 0.001) and the total cerebral volumes (*r* = 0.8, *p* < 0.001) were positively associated with gestational age, indicative of larger volumes at older gestational ages.

### Manual segmentation protocol validation: Intra-reliability test

The thalamus and cerebellum were re-segmented by a single rater (MM) to assess the consistency of the three-step manual segmentation protocol. Re-segmentations of both the left and right thalamus and cerebellum in these images were performed at least 6 months after the original segmentations were performed to minimize memory effects in the rater. The intra-reliability test results are listed in Table 3. The IQR of the median DSCs of cerebellar and thalamic segmentations were 0.78 and 0.6, respectively. The overall median DSC was 0.7.

**TABLE 3:** Intra-reliability test – Dice similarity coefficients.

	Dice similarity coefficients
Cerebellum	0.78 (0.7–0.8)
Thalamus	0.6 (0.5–0.7)
Overall	0.7 (0.5–0.7)

The median Dice similarity coefficients for cerebellar and thalamic segmentations, and both segmentations combined. IQR, interquartile range (25%ile–75%ile).

### Registration-based segmentation reliability test: Comparisons of dice similarity coefficients

**FIGURE 6:**
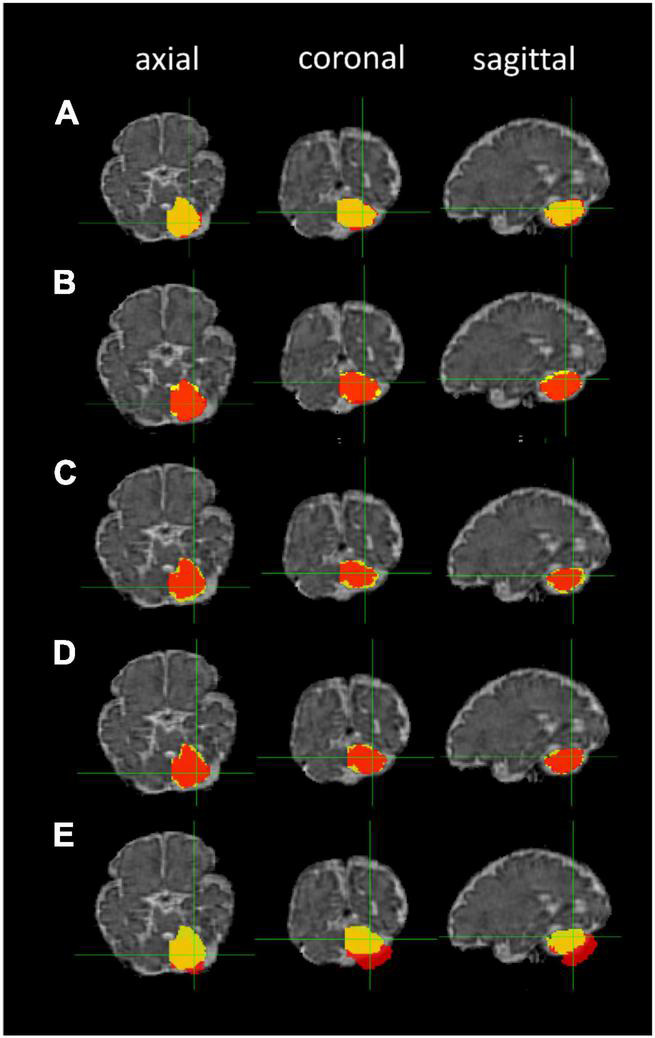
Cerebellar masks: registration-based segmentation (red) versus manual segmentation (yellow). The masks are shown in axial, coronal, and sagittal planes from left to right. Row **(A)** ANTs linear registration (MI); **(B)** ANTs linear registration (CC); **(C)** ANTs nonlinear registration (CC); **(D)** ANTs nonlinear registration (MI); and **(E)** FLIRT linear registration.

The ANTs- (5–10 h/dataset) and FLIRT-based (10 min/dataset) registrations of the 36-week GA fetal brain atlas into the native spaces of the individual fetal MR images were successfully processed in all participants. The median DSCs comparing the five image registration methods to the manual segmentation method were: (1) FLIRT linear registration (affine) using the correlation ratio similarity metric, (2) ANTs linear registration (rigid and affine) using the MI similarity metric (ANTs Lin MI), (3) ANTs linear registration using the CC similarity metric (ANTs Lin CC), (4) ANTs nonlinear registration (rigid, affine, and SyN) using the MI similarity metric (ANTs NL MI), and (5) ANTs nonlinear registration using the CC similarity metric (ANTs NL CC) for left and right cerebellum and thalamus segmentations. The cerebellar masks produced by the five registration methods using different similarity metrics are shown in Figure 6.

The median DSCs of the five registration methods for the cerebellum segmentations, thalamus segmentations, and both segmentations are listed in Table 4. Overall, the FLIRT linear registration resulted in non-optimal estimation with gross misalignment of the masks on the fetal MR image. The ANTs nonlinear registration (CC) had the highest median Dice similarity index. The ANTs non linear registration (MI) also demonstrated a very good performance.

**TABLE 4:** Median Dice similarity coefficients.

Registration method	Both	Cerebellum	Thalamus
FLIRT	0.54 (0.44–0.63)	0.62 (0.46–0.73)	0.52 (0.39–0.66)
ANTs Lin MI	0.70 (0.58–0.74)	0.80 (0.73–0.83)	0.59 (0.48–0.7)
ANTs Lin CC	0.72 (0.59–0.75)	0.80 (0.74–0.83)	0.61 (0.48–0.71)
ANTs NL MI	0.72 (0.63–0.76)	0.79 (0.75–0.83)	0.62 (0.49–0.68)
ANTs NL CC	0.74 (0.65–0.76)	0.79 (0.76–0.82)	0.65 (0.52–0.71)

The median Dice similarity coefficients and the interquartile ranges of the cerebellum, thalamus, and both subcortical segmentations using five registration methods compared to manual segmentations.

The median DSCs of both subcortical segmentations revealed that ANTs NL CC and ANTS NL MI were high with the linear registrations being comparable, while those produced by FLIRT were the lowest. The DSCs for the linear (i.e., ANTs rigid and affine, and FLIRT affine) and nonlinear (i.e., ANTs nonlinear with MI and CC similarity metrics) methods for the thalamus and cerebellum segmentations were compared using Friedman’s Repeated Measure Analysis of Variance by Ranks. Upon comparison of the left and right cerebellar DSCs (*n* = 50), the calculated mean ranks were significantly different from one another (df = 4, test statistic = 100.84, *p* < 0.001). *Post hoc* pairwise comparisons revealed that the mean ranks were significantly different for the FLIRT-based registrations compared to the ANTs linear and nonlinear methods (all, *p* < 0.001; Table 5). Additionally, none of the mean ranks differed for any of the ANTs based registration methods (all *p* > 0.88).

**TABLE 5:** *Post hoc* comparisons of mean ranks: fetal cerebellar segmentations.

Sample 1–sample 2	Standard test statistic	*P*-value*
**FLIRT – ANTs NL CC**	**6.77**	**<0.001**
**FLIRT – ANTs Lin MI**	**7.65**	**<0.001**
**FLIRT – ANTs NL MI**	**8.41**	**<0.001**
**FLIRT – ANTs Lin**	**−8.48**	**<0.001**
ANTs NL CC – ANTs Lin MI	0.89	0.9
ANTs NL CC – ANTs NL MI	1.64	0.9
ANTs NL CC – ANTs Lin	−1.71	0.88
ANTs Lin MI – ANTs NL MI	0.76	0.9
ANTs Lin MI – ANTs Lin CC	−0.82	0.9
ANTs NL MI – ANTs Lin CC	−0.06	0.9

Results of a Dunn’s pairwise *post hoc* tests on the mean ranks. *Bonferroni corrected for multiple comparisons. Significant values are in bold.

Subsequently, the DSCs produced by the linear and nonlinear registrations algorithms compared to the manual segmentations were examined for the left and right thalamic segmentations and were also significantly different (*n* = 50, df = 4, test statistic = 47.36, *p* < 0.001). Pairwise comparisons indicated slightly different results than seen for the cerebellar segmentations, whereby FLIRT-based registrations were associated with significantly different mean ranks compared to the ANTs-based nonlinear registration methods, including ANTs NL MI and NL CC, but also the ANTs Lin CC method (all *p* < 0.002; Table 6).

**TABLE 6:** *Post hoc* comparisons for mean ranks: fetal thalamic segmentations.

	Standard test statistic	*P*-value*
FLIRT – ANTs Lin MI	2.15	0.32
**FLIRT – ANTs Lin CC**	**−3.67**	**0.002**
**FLIRT – ANTs NL MI**	**4.49**	**<0.001**
**FLIRT – ANTs NL CC**	**6.45**	**<0.001**
ANTs Lin MI – ANTs Lin CC	−1.52	0.9
ANTs Lin MI – ANTs NL MI	2.34	0.19
**ANTs Lin MI – ANTs NL CC**	**−4.30**	**<0.001**
ANTs Lin CC – ANTs NL MI	0.82	0.1
ANTs Lin CC – ANTs NL CC	2.78	0.05
ANTs NL MI – ANTs NL CC	−1.96	0.5

Results of a Dunn’s pairwise *post hoc* tests on the mean ranks. *Bonferroni corrected for multiple comparisons. Significant values are in bold.

Comparison of the mean ranks indicated that ANTs NL CC performed significantly better than ANTs Lin MI (*p* < 0.001).

**FIGURE 7:**
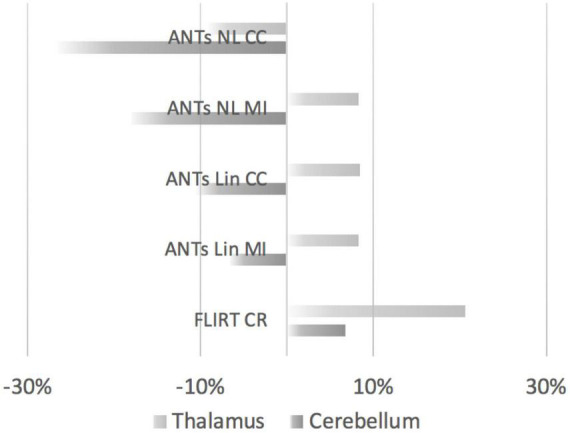
Comparisons of thalamic and cerebellar volumes produced by FLIRT- or ANTs-based methods relative to manually segmented volumes. The overlap (positive values indicate overestimation, negative values indicate underestimation) is displayed according to the registration methods from top to bottom. Top to bottom: ANTs nonlinear registration (CC, cross-correlation); ANTs nonlinear registration (MI, mutual information); ANTs linear registration (CC); ANTs linear registration (MI); and FLIRT linear registration.

We further compared the volumes extracted by the 5 registration methods relative to the manually segmented volumes. The extracted volumes for the cerebellum and thalamus based on the FLIRT and ANTs-based methods were subtracted from the manually segmented volumes. The differences in the volumes were then divided by the manually segmented volumes and the resulting values were converted to percentages (Figure 7). Overall, the cerebellar segmentations were more likely to be underestimated by ANTs-based methods. FLIRT-based registration of the thalamus and the cerebellum resulted in overestimation of the volumes.

## Discussion

Fetal MRI represents one of the next frontiers in clinical, translational and basic science research, not only to improve our understanding of the developing fetal brain, but to aid in early diagnosis, particularly for fetuses at-risk for adverse neurodevelopmental outcomes (Andescavage et al., 2017; Mufti et al., 2021; Rajagopalan et al., 2021). The study of the brain and other organs in the fetus has been limited to primarily non-invasive ultrasound technology. While ultrasound offers many advantages due to its low cost and ease of use in hospital settings, it is limited in terms of its spatial resolution to study fetal brain structure. MRI of the fetal brain offers superior 3D image resolution and can be used to study brain volumetric development.

This work aimed to develop a semi-automatic pipeline to segment fetal brain volumes acquired in third-trimester images. A recently developed deep learning algorithm was employed to mask the fetal brain and reconstruct MR images in second-trimester fetuses. Analyzing fetal MR images using brain segmentation toolkits designed for adult populations is impractical due to the presence of motion artifacts from fetal movements. This study aimed to overcome this obstacle in fetal MRI by applying segmentation, volumetric reconstruction, and image normalization toolkits to build a semi-automated process for fetal brain subcortical segmentation in T2-weighted fetal MR images that were acquired during the third trimester of pregnancy.

The fetal brain was masked in three anatomical 2D planes (axial, sagittal, and coronal) in the first step. Then the segmented 2D fetal brains and brain masks in three planes were reconstructed into 3D brain volumes and masks. After skull-stripping and orientation tag correction, linear and nonlinear image registration methods were evaluated in terms of their accuracy in segmenting cortical and subcortical structures by applying an age-appropriate MRI atlas. In turn, the subcortical labels of the chosen atlas were aligned with the individual fetal MR images using two different image registration toolkits (ANTs and FLIRT) using linear (ANTs Lin MI/CC and FLIRT) and nonlinear registration methods (ANTs NL MI/CC). The optimal cortical and subcortical segmentation performance was determined by applying and comparing two image registration toolkits for both nonlinear and linear image registration algorithms with different configurations of similarity metrics. The aligned subcortical labels were then compared with manually segmented thalamus and cerebellum subcortical masks. The manually labeled masks were considered ground truth for later comparison with the atlas-based registration. The nonlinear registration methods within ANTs provided improved results compared to a linear transformation (FLIRT) for the cerebellum segmentations as well as in comparison to the linear methods within ANTs, primarily for the thalamic segmentations. The ANTs MI and CC similarity metrics are optimized in terms of translation, rotation, scaling, and shearing during the registration of the images. Nonlinear registration methods, while computationally more intensive, may be more suitable for small samples of fetal brain images acquired during the third trimester to have higher quality results. Overall, our findings indicated that ANTs-based nonlinear registration methods using the MI and CC similarity metric performed adequately and may be more practical for processing larger datasets but with additional computational processing time.

### Semi-automatic registration-based fetal subcortical segmentation

This research utilized a machine learning-based segmentation algorithm from the NiftyMIC toolkit (Ebner et al., 2020) to significantly mitigate motion artifacts, segment the fetal brain images acquired during the third trimester in 2D, and reconstruct 2D images in three planes into 3D volumes. The NiftyMIC toolkit (Ebner et al., 2020) is open-source, Python-based software for research within the Guided Instrumentation for Fetal Therapy and Surgery (GIFT-Surg) project, which is an international research consortium focused on developing technology, tools and training to facilitate fetal surgery (Joyeux et al., 2018). The software can reconstruct an isotropic, high-resolution brain volume from multiple low-resolution 2D image slices acquired in fetuses. The NiftyMIC 2D segmentation was originally trained and developed for second-trimester fetal MR images. This masking step is essential for the remaining workflow steps. In the current work, each fetal mask required visual inspection and manual editing to aid the performance of the automatically generated labels. With the adequate 2D fetal brain masks serving as input, the NiftyMIC volumetric reconstruction process performed smoothly. Overall, NiftyMIC performed well on most images, and the performance was comparable to what was published in second-trimester images.

Machine learning algorithms are known to theoretically perform well to learn and predict data patterns when the process is trained with enough data (Cardenas et al., 2019). This performance depends on the problem’s complexity and the sophistication of the machine learning algorithm. The NiftyMIC 2D brain segmentation of third-trimester fetal MR images did not always perform reliably on the third-trimester MRI data. The NiftyMIC (*niftymic_segment_fetal_brains*) machine learning algorithm was originally trained with MR images of second-trimester healthy fetuses and fetuses diagnosed with spina bifida. Exponential growth of the fetal brain from the second to the third trimester results in significant cortical and subcortical morphology changes.

Additionally, during the third trimester, the fetal brain becomes increasingly myelinated (Dubois et al., 2008a,b, 2014; Wilson et al., 2021). Structural MR images weighted by T1 or T2 relaxation times will be influenced by different water and fat contents in the fetal brain compared to that seen in adults. This difference results in different signal intensities in the voxels of MR images of the fetal brain, which can vary in fetuses even compared to 6-month-old infants due to the rapid changes in overall growth and myelination (Dubois et al., 2014). Less is known about tissue intensity changes between second-trimester and third-trimester fetuses; however, in relation to the current work, the image intensity of the voxels of the gray and white matter tissues of the training data used for NiftyMIC may have been quite different from that of our third-trimester data. These factors could have notably influenced the machine learning algorithm’s performance.

The linear and nonlinear registration algorithms paired with various similarity metrics were successfully applied to register the labeled atlas into native space for cortical and subcortical segmentation of the MRI scans acquired in third-trimester fetuses. The use of different similarity metrics applied to fetal deep-brain segmentation was explored. The registered thalamic and cerebellar masks were compared to manually segmented masks. The ANTs nonlinear registration tool (Avants et al., 2008) reliably segmented deep brain structures of fetal brains on MR images for both the cerebellum and thalamus. The DSCs of ANTs Lin CC indicated a good agreement between the atlas-based and manual segmentations. The ANTs Lin MI registration had similar DSCs for both thalamic and cerebellar segmentations. According to the guidelines for interpreting DSCs (Cohen, 1960), the median DSCs of ANTs Lin MI and CC indicated a substantial agreement between the registration-based semi-automatic segmentation and manual segmentation for estimating fetal deep brain structures. However, there was a notable performance difference between thalamic and cerebellar segmentations using all five registration methods. The median DSCs of the thalamic segmentations were lower than that of the cerebellar segmentations, which indicated very good agreement (>0.7) and only moderate agreement (0.5–0.6) between the registration-based and manual thalamic segmentations using ANTs Lin MI and CC. Findings indicated that ANTs-based nonlinear image registration did not outperform ANTs-based linear image registration for segmenting the cerebellar structures. The CC similarity metric, suitable for intra-modality MR image normalization, was sufficient for our fetal MRI data. The additional calculations involving histogram matching from the MI metric did not substantially improve the image registration quality. Therefore, using the CC metric, which requires less computation time to register the data, is sufficient for processing datasets, particularly those with larger sample sizes.

This semi-automatic fetal subcortical segmentation method may be very beneficial for future studies of fetal neurodevelopment. The *in utero* origin of neurodevelopmental delay reflected in smaller cortical and subcortical volumes can be studied by applying this methodology to a larger fetal MR image dataset that has the potential for significant savings in terms of time and labor devoted to manual segmentations. The whole brain volume and deep brain structures such as the hippocampus are important for learning and memory processes and can be segmented from the MR images for comparison, analysis and developmental outcome prediction (Eichenbaum, 1997; Bird and Burgess, 2008; Milivojevic and Doeller, 2013). The proposed methodology could also be utilized to study second-trimester fetal volumetric development. From the second to the third trimester, fetal neurodevelopment could be monitored by segmenting and calculating subcortical and brain growth in high-risk groups. This method could potentially reveal when the variations in brain morphology occur to aid in the early diagnosis of fetal brain abnormalities in clinical settings.

The ANTs-based nonlinear image registration performed slightly better than the ANTS-based linear image registration for aligning the fetal brain atlas to our dataset’s native MR image space. However, this difference was not strongly evident statistically. The amount of deformation of the image when warping the atlas might have been minimal, given that the difference in the shape of the fetal brain of the atlas and our acquired fetal MR images was comparable in terms of the anatomy. Linear mislocalization of the fetal brains between the atlas image and the target image may have contributed to spatial differences. The ANTs-based nonlinear registration is more time-consuming than linear registration, with a higher requirement of computation abilities while providing reliable subcortical segmentation performance.

## Conclusion

Antenatal development of the fetal cortex and subcortical structures is a complex neurophysiological process. The development of the nervous occurs through genetically predetermined events, including cellular proliferation, neuronal migration, and differentiation of cells into specialized subtypes, followed by synaptogenesis, which provides the formation of cortical and subcortical circuitry. Environmental influences such as maternal diet and even stress can alter these processes and, in more severe cases, can lead to growth restriction of the fetus. The study of fetal brain development using volumetric MRI provides a window into the development of the cortex and subcortical structures in typical and atypically developing fetuses. This work developed and evaluated a semi-automatic pipeline to segment the cortex and subcortical structures in third-trimester images. A novel deep learning-based algorithm was used to segment and reconstruct 3D MR images of the entire fetal brain. An atlas to segment cortical and subcortical structures was aligned to the fetal brain images. Five registration algorithms were compared to gold-standard manual segmentations of subcortical structures. Overall a deformable registration method, ANTs using a CC metric provided optimal performance to segment the cortical structures, and may be favorable for large datasets or for use in high-resource settings without access to high throughput computational facilities. Future work, using deep-learning methods for image registration and segmentation may facilitate more automated methods for cortical and subcortical parcellation in the fetus. Larger datasets with wider gestational age ranges would aid in facilitating artificial intelligent approaches to fetal brain development. Additionally, applying this atlas-based method to study deep-brain macrostructural development in high-risk fetuses would be a future step. Fetal brain growth is a key marker for developmental outcomes. Methods to characterize subcortical development in typically and atypically fetuses could aid in the detection of potential biomarkers associated with delayed or arrested growth. Utilizing multi-modal MR methods may also further facilitate fetal brain tissue extraction.

## Data availability statement

The raw data supporting the conclusions of this article will be made available by the authors upon reasonable request.

## Ethics statement

The studies involving human participants were reviewed and approved by the Health Sciences Research Ethics Board at Western University. The participants provided their written informed consent to participate in this study.

## Author contributions

JW, EN, MM, BV, RE, CM, SR, and ED were involved in the study design, database variable creation, test material selection, and data acquisition design and execution of the data analytic strategy, and reviewed and revised the final version of the manuscript. JW, EN, SR, and ED conceptualized the execution of the data analytic strategy, contributed to the data analysis, and reviewed and revised the final draft of the manuscript. All authors approved the final manuscript as submitted and agree to be accountable for all aspects of the work.

## Footnotes

1
https://fsl.fmrib.ox.ac.uk/fsl/fslwiki/FSLeyes

2
http://itksnap.org

## Abbreviations

ANTs: advanced normalization tools
ANTs Lin MI: ANTs linear registration with mutual information similarity metric
ANTs Lin CC: ANTs linear registration with cross-correlation similarity metric
ANTs NL MI: ANTs nonlinear registration with mutual information similarity metric
ANTs NL CC: ANTs nonlinear registration with cross-correlation similarity metric
CSF: Cerebrospinal Fluid
CNN: convolutional neural network
FGR: fetal growth restriction
FLIRT: FMRIB’s Linear Image Registration Tool
FSE: fast spin echo
GUI: graphical user interface
IUGR: Intrauterine Growth Restriction
MRI: magnetic resonance imaging
TR: repetition time
TE: echo time
ROI: regions of interest
SyN: symmetric image normalization.
